# 

**Published:** 1844-10-01

**Authors:** 


### BIBLIOGRAPHICAL RECORD.

1. A Practical Treatise on Midwifery. By M. Chailly, M.D. Illustrated with Plates. Translated from the French, by Gunning S. Bedford, M.D. New York, 1844.

2. Defence of Hahnemann and his Doctrines; including an Exposure of Dr. Alexander Wood's "Homœopathy unmasked." Octavo, pp. 92. Baillière, 1844.

3. The Plagiarisms of Julius Jeffreys, Esq. F.R.S. By G. Calvert Holland, M.D. Churchill—London, June 1844.

4. A Practical Treatise on Diseases of the Eye. By William Jeaffreson, Esq. 8vo, pp. 307. Renshaw—London, 1844.

5. On the Decrease of Disease effected by the Progress of Civilization. By C. F. H. Marx, M.D. and R. Willis, M.D. Octavo, pp. 102. Longman and Co., July 1844.

6. Graefenberg:—or a True Report of the "Water-Cure," with an Account of its Antiquity, &c. By Robert Hay Graham, M.D. Octavo, pp. 232. Longman and Co., 1844.

7. The Chemical and Physiological Balance of Organic Life:—an Essay, by M. J. Dumas and M. J. B. Boussingault. The Third Ed. with New Discoveries. Octavo, pp. 152. Baillière—London, 1844.

8. Manual of the Practice of Medicine:—the Result of Fifty Years' Experience. By W. C. Hufeland, M.D. From the 6th edition of the German. Translated by C. Bruckhausen, M.S. & R. Nelson, M.D. Baillière—London, June 1844.

9. The Northern Journal of Medicine (Monthly). By Drs. Seller and Kemp. No. 3, July 1844. Edinburgh—Oliver and Co. Price 1s. 6d.

10. The Actual Process of Nutrition in the Living Structure demonstrated by the Microscope, and the Renewal of the Tissues and Secretions, &c. &c. (Second Series of Experiments). By Wm. Addison, F.L.S. Churchill—London, 1843.

11. Sequel to "Homœopathy unmasked." By Alexander Wood, M.D., Edin. D. Bogue, London.

12. Modern Surgical Instruments, chiefly of France and Germany, illustrated. Henry Renshaw, Strand, London, 1844.

13. A History of British Fossil Mammalia and Birds. By Richard Owen, F.R.S. Hunterian Professor, &c. With numerous illustrative Engravings. Van Voorst, London, February 1844.

14. Second Report of the Medical Benevolent Fund Society of Ireland, for the year ending May 1844.

15. Narrative of a Voyage to Madeira, Teneriffe, and along the Shores of the Mediterranean, including Algiers, Egypt, &c. &c. By W. R. Wilde, M.R.I.A. Second Edition, enlarged and revised. W. Curry and Co., Dublin; Longman and Co., London. 1844.

16. The Dispensing Chemist's and Medical Pupil's Assistant;—containing Latin Directions, with their Translations, &c. &c. By John F. Burke, M.R.C.S.

☞ *A useful little Compilation.*

17. The Northern Journal of Medicine; a Monthly Survey, &c. &c. No. 4, August 1844. Conducted by Drs. Seller and Kemp. Edinburgh, 1844.

18. Reports of the Pennsylvania Hospital for the Insane, &c. &c. By Thomas S. Kirkbride, M.D. Philadelphia, 1844.

19. The Medical Examiner, and Retrospect of the Medical Sciences. By Dr. Clymer. Philadelphia, Dec. 1843.

20. The Educational and Subsidiary Provisions of the Birmingham Royal School of Medicine and Surgery. By the Rev. V. Thomas, B.D

21. Report of the Meeting of the Queen's College, Birmingham.

22. Researches into the Physical History of Mankind. By JAMES COWLES PRITCHARD, M.D. Vol. IV., 3rd Edition. Sherwood & Co., London. 1844.

23. The Medical Examiner, and Record of Medical Science. Edited by Dr. R. M. HUSTON. Nos. 11, 12, 13, 14, and 15. Philadelphia, 1844.

37. The New York Journal of Medicine and the Collateral Sciences. Edited by SAMUEL FORRY, M.D. July, 1844. New York.

25. The American Journal of Insanity. Edited by the Officers of the New State Lunatic Asylum. Utica, 1844.

26. Transactions of the New York State Medical Society. Vol. VI. Part I. Albany, 1844.

